# Trapezius transfer to treat flail shoulder after brachial plexus palsy

**DOI:** 10.1186/1749-7221-2-2

**Published:** 2007-01-12

**Authors:** Ricardo Monreal, Luis Paredes, Humberto Diaz, Pastor Leon

**Affiliations:** 1 Manuel Fajardo Teaching Hospital. Orthopedics and Traumatology Department, Zapata y calle D, Vedado, CP:10400, Havana, Cuba

## Abstract

**Background:**

After severe brachial palsy involving the shoulder, many different muscle transfers have been advocated to restore movement and stability of the shoulder. Paralysis of the deltoid and supraspinatus muscles can be treated by transfer of the trapezius.

**Methods:**

We treated 10 patients, 8 males and 2 females, by transfer of the trapezius to the proximal humerus. In 6 patients the C5 and C6 roots had been injuried; in one C5, C6 and C7 roots; and 3 there were complete brachial plexus injuries. Eight of the 10 had had neurosurgical repairs before muscle transfer. Their average age was 28.3 years (range 17 to 41), the mean delay between injury and transfer was 3.1 years (range 14 months to 6.3 years) and the average follow-up was 17.5 months (range 6 to 52), reporting the clinical and radiological results. Evaluation included physical and radiographic examinations. A modification of Mayer's transfer of the trapezius muscle was performed. The principal goal of this work was to evaluate the results of the trapezius transfer for flail shoulder after brachial plexus injury.

**Results:**

All 10 patients had improved function with a decrease in instability of the shoulder. The average gain in shoulder abduction was 46.2°; the gain in shoulder flexion average 37.4°. All patients had stable shoulder (no subluxation of the humeral head on radiographs).

**Conclusion:**

Trapezius transfer for a flail shoulder after brachial plexus palsy can provide satisfactory function and stability.

## Background

After severe brachial palsy involving the shoulder, secondary operations are sometimes required to restore function. These include shoulder artrhodesis, rotational osteotomy, muscle transfer or a combination of these techniques.

For paralysis of the deltoid and supraspinatus muscle many different muscle transfers have been advocated to restore movement and stability of the shoulder. These include transfer of the trapezius, pectoralis major and teres major, latissimus dorsi, and combined biceps and triceps.

In a classic monograph; Saha [1] gave details of his experience with transfer of the trapezius, using a modification of the technique originally described by Bateman [2]. However, the absence of clear indications for the operation and expecting too much for this transfer alone has led to its infrequent use.

We have evaluated the results of the trapezius transfer for flail shoulder after brachial plexus injury.

## Methods

We treated 10 patients, 8 males and 2 females, by transfer of the trapezius to the proximal humerus. In 6 patients the C5 and C6 roots had been injured; in one C5, C6 and C7 roots; and in 3 there were complete brachial plexus injuries. Eight of the 10 had had neurosurgical repairs before muscle transfer.

Their average age was 28.3 years (range 17 to 41), and the average follow-up was 17.5 months (range 6 to 52). The mean delay between injury and transfer was 3.1 years (range 14 months to 6.3 years).

All patients had elbow flexion (2 had had previous Steindler flexorplasties) and 6 patients had good ipsilateral hand function.

Evaluation included physical and radiographic examinations. The active abduction/flexion shoulder motion was recorded (power between 3 to 5 grades according to MRC scale). Shoulder abduction was measured as the angle between the trunk and the arm. The pre-operative average was 3.1° (range 0° to 30°). The average shoulder forward flexion was 4.5° (range 0° to 45°). In all patients, the deltoid, supraspinatus, teres minor, infraspinatus and subscapularis were paralysed and the trapezius, levator scapulae were preserved. The rhomboids were affected in 2 patients. Paralysis of deltoid and supraspinatus was confirmed by EMG. All patients were unemployed at the time of trapezius transfer. Radiological subluxation of the shoulder was present in all cases. The subjective assessment of the patients was not considered.

Surgery can be considered if the patient presents flail shoulder at more than one year after the accident without spontaneous recovery or when it is clear that recovery following neurosurgical repair is not progressing any more. A simple trapezius transfer is compatible with the later return of some function to other shoulder girdle muscles. Passive shoulder abduction of 80° is an important pre-requisite before transfer. The only contra-indication is advanced degeneration of the shoulder.

A modification of Mayer's [3] transfer of the trapezius muscle was performed in which a portion of the acromion is removed to allow for a more straight-line pull. The lateral aspect of the acromion and its attached trapezius is removed, and its undersurface is roughened with a rasp. Fixation with one or two screws secures the acromion and trapezius transfer to the proximal part of the humeral shaft.

The principal goal of this work was to evaluate the results of the trapezius transfer for flail shoulder after brachial plexus injury.

### Surgical technique

The patient is placed supine with a sand-bag under the shoulder. The shoulder, the neck, and the whole arm are prepared and free.

A saber-cut incision is made from the inferior border of the anterior axillary fold over the anterior aspect of the shoulder to a point a few centimetres lateral to the medial border of the scapula and just distal to the scapular spine. The deltoid origin is then cut from the lateral third of the clavicle, the acromion, and the lateral half of the spine of the scapula.

A Gigli wire saw is used to transect the root of the acromion, and then the lateral clavicle, so as to separate the lateral 1 cm of the clavicle with the acromion. The remaining insertions of the trapezius are elevated from the clavicle and the scapular spine to 2 cm from the vertebral border of the scapula. Careful dissection is needed to define the interval between the trapezius and the supraspinatus. Special attention is needed to preserve the neurovascular bundle of the spinal accessory nerve and transverse cervical artery, which courses from deep to superficial through the trapezius.

The partly detached deltoid is split longitudinally to expose the proximal humerus, which is scored with an osteotome. The arm is then abducted to 90°, and the acromiocalvicular fragment with its trapezius insertion is fixed to the humerus with two screws, ensuring firm bone-to-bone. The wound is thoroughly irrigated with saline solution, and the deltoid is sutured on top of the new trapezius insertion. The skin is closed in two layers over suction drains a shoulder spica applied with the shoulder in 90° of abduction.

Postoperative management. Drains are removed on the second or third day. The spica is worn for six weeks or until union is seen between the acromion fragment and the humerus. The arm is then allowed to adduct progressively and a vigorous physical therapy programme is started. As strength improves, more resisted muscle strengthening exercises are added.

## Results

**Figure 1:**
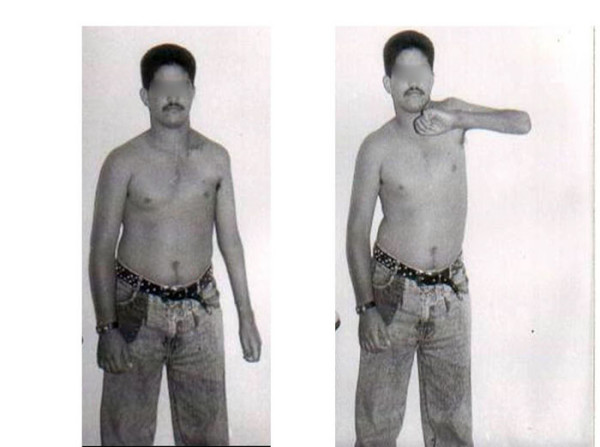
A 18-year-old man 16 months after trapezius transfer on the left side, showing 90° of abduction.

**Figure 2:**
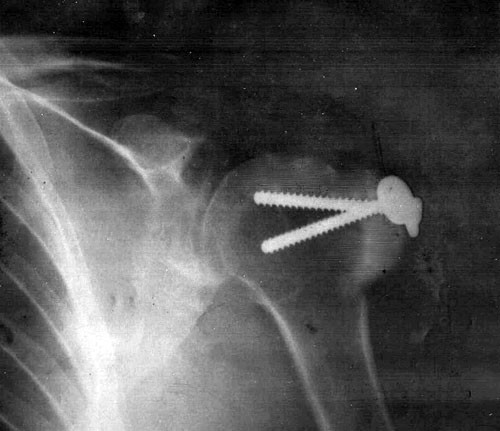
The radiograph shows that there is not downward subluxation of the humeral head.

The transfer improved function of the shoulder (Figure 1). Postoperatively, the average gain in shoulder abduction was 46.2° (p < 0.001, Fisher exact test); the gain in shoulder flexion average 37.4° (p < 0.001). All patients had stable shoulders (no subluxation of the humeral head on radiographs, Figure 2).

Surgical time averaged 2 hours (range 1 to 4), and the estimated mean blood loss was 200 ml. There were no postoperative complications.

## Discussion

Severe injuries to the brachial plexus cannot always be successfully repaired; even failures are seen after the best repair. Unsatisfactory or incomplete results affect abduction, external rotation and forward projection of the humerus at shoulder level.

Flail shoulder secondary to a brachial plexus injury is difficult to treat. After neurosurgical treatment and adequate physiotherapy, reconstructive surgery may be needed to improve the stability and function of the shoulder.

Deltoid and supraspinatus paralysis may be managed by shoulder fusion [4-6] or muscle transfer [7]. Shoulder arthordesis has been considered the procedure of choice in patients with flail shoulder after brachial plexus palsy, but is irreversible and has a high complication rate. Cofield and Briggs [8] pointed out the disadvantages of arthrodesis (24% incidence of fractures, 25% had no improvement and 15% had aggravation of pain).

Trapezius, levator scapulae and rhomboid muscles remain healthy or recover in 96% of cases, therefore are available for transposition.

Several muscle transfers have been advocated to restore movement and stability of the shoulder after poliomyelitis [7,9,10], and, more recently, the use of these procedures after brachial plexus palsy has been reported. [11-14]

Aziz, Singer and Wolff [12] discuss trapezius transfer for flail shoulder after brachial plexus palsy, finding it a simple procedure with minimal blood loss, which provided functional improvement.

Passive shoulder abduction of 80° is an important pre-requisite, and requires intensive physiotherapy before transfer. If 80° is not obtained, shoulder arthrodesis is recommended [13].

Trapezius transfer to treat flail shoulder after a brachial plexus injury will allow the patient to position the arm much better, even when functional recovery is not adequately strong to keep the shoulder stable. The procedure is relatively simple with minimal blood loss and the only contraindication is advanced degeneration of the shoulder. Trapezius transfer can be used combined with other transfers to achieve optimal use of the upper limb.

## Conclusion

Trapezius transfer can provide satisfactory functional improvement and it is better than arthrodesis for paralysis of the shoulder after brachial plexus injury.
